# Measuring the ICF components of impairment, activity limitation and participation restriction: an item analysis using classical test theory and item response theory

**DOI:** 10.1186/1477-7525-7-41

**Published:** 2009-05-07

**Authors:** Beth Pollard, Diane Dixon, Paul Dieppe, Marie Johnston

**Affiliations:** 1 School of Psychology, University of Aberdeen, Aberdeen, AB24 2UB, UK; 2 Department of Psychology, University of Stirling, Stirling, FK9 4LA, UK; 3 Peninsula College of Medicine and Dentistry, University of Plymouth, Plymouth, PL4 8AA, UK

## Abstract

**Background:**

The International Classification of Functioning, Disability and Health (ICF) proposes three main health outcomes, Impairment (I), Activity Limitation (A) and Participation Restriction (P), but good measures of these constructs are needed The aim of this study was to use both Classical Test Theory (CTT) and Item Response Theory (IRT) methods to carry out an item analysis to improve measurement of these three components in patients having joint replacement surgery mainly for osteoarthritis (OA).

**Methods:**

A geographical cohort of patients about to undergo lower limb joint replacement was invited to participate. Five hundred and twenty four patients completed ICF items that had been previously identified as measuring only a single ICF construct in patients with osteoarthritis. There were 13 I, 26 A and 20 P items. The SF-36 was used to explore the construct validity of the resultant I, A and P measures. The CTT and IRT analyses were run separately to identify items for inclusion or exclusion in the measurement of each construct. The results from both analyses were compared and contrasted.

**Results:**

Overall, the item analysis resulted in the removal of 4 I items, 9 A items and 11 P items. CTT and IRT identified the same 14 items for removal, with CTT additionally excluding 3 items, and IRT a further 7 items. In a preliminary exploration of reliability and validity, the new measures appeared acceptable.

**Conclusion:**

New measures were developed that reflect the ICF components of Impairment, Activity Limitation and Participation Restriction for patients with advanced arthritis. The resulting Aberdeen IAP measures (Ab-IAP) comprising I (Ab-I, 9 items), A (Ab-A, 17 items), and P (Ab-P, 9 items) met the criteria of conventional psychometric (CTT) analyses and the additional criteria (information and discrimination) of IRT. The use of both methods was more informative than the use of only one of these methods. Thus combining CTT and IRT appears to be a valuable tool in the development of measures.

## Aim

The aim of this paper was to develop measures that reflect the health components identified by the International Classification of Functioning, Disability and Health (ICF) for use with people having joint replacement surgery. Item analysis was carried out using both Classical Test Theory (CTT) and Item Response Theory (IRT) on a group of candidate Impairment (I), Activity Limitation (A) and Participation Restriction (P) items. The items had been previously judged to be measuring one, and only one, of the three ICF components [1].

## Background

**Figure 1:**
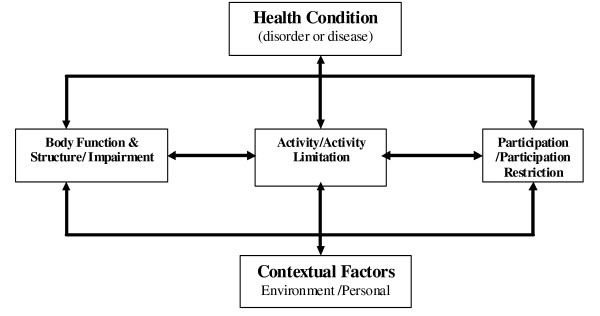
**The ICF model**.

The dominant theoretical models of health outcomes or the consequence of disease have been the models developed by the World Health Organisation [2,3]. The most recent version, the International Classification of Functioning, Disability and Health (ICF [2]) is based on a biopsychosocial model that integrates medical and social models (Figure 1). The ICF model identifies three main distinct constructs (components), Impairment (I), Activity Limitation (A) and Participation Restriction (P) and their respective opposites, Body Function and Structure, Activity and Participation [2].

In developing measures of these constructs, it is important to ensure that the measures assess *only *the construct of interest and are not simultaneously measuring other constructs within the model or outwith the model. If measures are not 'pure' (i.e. only measuring the construct of interest), empirical evidence for relationships between constructs in the model may be misleading. Thus, it is possible, that significant correlations between constructs, and support for models may be due not to true relationships and the validity of the model, but to the overlap of constructs within the measures. It is also possible that a lack of relationship between constructs may also be due to contaminated measures. Hence, only if we can establish distinct measures of the main ICF constructs can we explore the relationships between these constructs and attempt to progress to a truly testable theoretical model. Contaminated measures may also mask positive or negative effects of interventions.

With the wide acceptance of the ICF framework, attempts have been made to link existing measures to ICF constructs and categories [1,4-7]. These studies have shown that the selected existing measures do not map onto single ICF constructs. Hence, there is a need for pure measures of the ICF constructs. Very few measures have been developed based on the ICF constructs for use with people having joint replacement although a measure for people with knee OA has been developed but specifically to reflect Japanese culture [8]. Additionally, a measure of participation restriction for use in population studies has been developed based on the ICF [9] and recently a measure of participation has been developed for OA but it was not based on the ICF [10].

We have previously shown that existing measures used to assess health status in people with osteoarthritis (OA) cannot be used to uniquely measure the ICF constructs of Impairment (I), Activity Limitation (A) and Participation Restriction (P) [1]. However, application of the method of Discriminant Content Validation [1,11] by expert judges identified a pool of pure I, A and P items within existing measures (i.e. items judged to be uncontaminated with other constructs in the ICF model) [1]. This pool of items may form the basis of new pure measures of I, A and P but further work needs to be done to select items from the pool for each measure to lessen the burden to patients and to eliminate redundant or misfitting items.

In an item analysis, the candidate items are completed by participants from the target population and analysed statistically. This analysis can suggest items that may not be appropriate for the measure that is required, and so may be removed from the item pool.

The Classical Test Theory (CTT) approach to item analysis is based on correlational data and the procedures usually involve maximising Cronbach's alpha [12] and selecting items with high factor loadings using exploratory factor analysis [13]. However, these methods have known limitations such as resulting in measures only tapping a small part of the underlying construct [14-16]. Additionally, and importantly, CTT methods are dependent on the sample and the set of items that the participants respond to

The newer methods of Item Response Theory (IRT) can provide additional information to CTT methods [17] and allow for the examination of individual items in more detail than CTT. The method has three big advantages, firstly, that within sampling error, the item parameters are not dependent on the ability levels of the sample i.e. they are sample invariant. Secondly, the score achieved by an individual is independent of the particular sample of items that the individual responds to [18]. Third, IRT gives indices of the informatic contribution of items, allowing the removal of redundant or non-discriminating items. IRT models are probabilistic and model respondents' response to an item, to a position on an underlying unidimensional hypothesised construct. Using IRT, estimates can be provided of both the items' discriminating ability and difficulty.

IRT also provides information functions, these indicate where an item is most useful on the underlying construct. The shape of an item information function is a combination of the item's discriminating ability and its difficulty. The item information function allows for the reliability of a measure to be explored throughout the entire underlying construct. In contrast, CTT only gives a single overall reliability estimate (Cronbach's alpha). Low information functions may indicate that an item may not be appropriate. This may be due to either the item not measuring the same thing as other items in the scale or the item being too difficult, poorly worded or out of context within the questionnaire [19].

The individual item information functions can be summed to form the test information function. This can indicate if there are areas on the underlying construct not covered by the selected items. If this is found, then new items may be written to cover these areas where the measure has low reliability.

Typically, item analysis has been carried out using CTT *or *IRT. CTT has been the standard method of item analysis and has been a valuable tool over many years [20]. However, CTT depends on the nature and size of the sample and the nature and number of items as well as having other limitations.

IRT can overcome many of the problems of CTT but is more difficult to perform and understand [20] and has less established guidelines. Hence, it has been suggested that the use of both methods may be more informative than only using a single method [19,20].

In this study, CTT and IRT methods were used independently to identify items that may be removed from the item pool. The item analysis was carried out for I, A and P separately; resulting in the exclusion of items from the pool. The relevant information from both methods was then combined and discrepancies examined.

## Method

### Design

A geographical cohort of participants from the Tayside Joint Replacement (TJR) cohort about to undergo hip or knee joint replacement surgery at Ninewells Hospital, Dundee were invited to complete assessments including pure I, A and P items. Data were analysed using CTT and IRT methods to identify appropriate items for I, A and P measures.

### Procedure

Ethics approval was obtained from the Tayside Committee on Medical Research Ethics. A questionnaire pack was sent to each participant's home approximately four weeks prior to surgery by the pre-operative assessment nurse at the hospital. The questionnaire pack consisted of an invitation to participate, patient information sheet, consent form, questionnaire and stamped return envelope. The participants completed the questionnaire at home and returned it by post to the research team.

### Participants

The questionnaire was sent to 1145 patients having their first hip or knee replacement on that particular joint and completed by 524 patients (43% response rate). Seventeen patients were excluded from the analysis as they completed the questionnaire on or after their scheduled operation date and 25 patients were excluded as they had an unknown operation date or did not record the date on which they completed the questionnaire. This resulted in a sample of 482 patients (who completed the questionnaire, on average, 34 days before surgery). The sample comprised 53% women and 55% were having hip replacements. The patients' mean age was 68.78 (s.d. = 9.9).

There were 25 patients whose diagnosis was not recorded. Of the remaining 457 patients, 93.4% had a diagnosis of osteoarthritis.

There was no difference in mean age or proportion of men to women between the responders and non-responders (i.e. those who did or did not agree to take part in the study and return the postal questionnaire). There was also no difference between responders and non-responders in terms of disease severity as measured by either the American Knee Score [21] (function and score) or on the Harris Hip score [22] which were the routine measures being used to assess all patients health status prior to surgery

### Measures

#### Pure measures

A pool of pure items was previously identified using Discriminant Content Validation by expert judges from 13 existing OA health outcome measures [1]. The items originated from the American Knee Score, Arthritis Impact Measurement Scale (AIMS, [23]), Disease Repercussion Profile (DRP, [24]), EuroQol [25], Functional Limitation Profile (FLP, [26]), Harris Hip score [22], Health Assessment Questionnaire (HAQ [27]), Lequesne Hip and Knee Indices [28], London Handicap Scale (LHS [29]), Oxford Hip and Knee Questionnaires (OXFORD [30,31]), RAND 36 item Short Form Health Survey (SF-36 [32]), Western Ontario and MacMaster Universities Osteoarthritis Index (WOMAC [33]), World Health Organisation Quality of life Assessment-Brief (WHOQOL [34]).

The pool of pure items comprised 74 I, 88 A and 44 P items. An initial procedure was necessary to eliminate items with overlapping content and reduce patient burden. This procedure resulted in 13 I, 26 A and 20 P candidate items (for details of this procedure and format of items see Additional file 1: initial item pool reduction). For all items a high score implies high limitation. Each item and its origin are in Tables 1, 2 and 3.

**Table 1:** I_ctt items ordered by difficulty

**Item**	**Origin**	**Mean**	**s.d.**
I1. Does remaining standing for 30 minutes increase your pain?	LEQUESNE	4.21	0.98

**I2. What degree of difficulty do you have bending and rotating your affected joint?**	**HARRIS**	**3.87**	**0.90**

I3. How would you describe the pain you usually have from your joint?	AIMS	3.86	0.66

I4. How often have you had severe pain from your arthritis?	AIMS	3.74	0.90

I5. How active has your arthritis been?	AIMS	3.74	0.83

I6. Have you been troubled by pain from your joint in bed at night?	OXFORD	3.68	1.21

I7. How severe is your stiffness after first wakening in the morning?	WOMAC	3.39	0.88

**I8. How severe is your stiffness after sitting, lying or resting later in the day?**	**WOMAC**	**3.26**	**0.80**

I9. How long has your morning stiffness usually lasted from the time you wake up?	AIMS	3.22	1.07

**I10. Has pain from your joint kept you awake during your night-time sleep?**	**STEERING GROUP**	**3.19**	**1.22**

I11. Have you felt that your knee or hip might suddenly 'give way' or let you down?	OXFORD	2.99	1.02

**I12. How often have you had pain in two or more joints at the same time?**	**AIMS**	**2.92**	**1.15**

113. Have you had any sudden, severe pain – 'shooting', 'stabbing' or 'spasms' – from the affected joint?	OXFORD	2.90	0.88

Items in bold removed by CTT/IRT item analysis

**Table 2:** A_ctt items ordered by difficulty

**Item**	**Origin**	**Mean**	**s.d.**
A1. What degree of difficulty do you have climbing up and down several flights of stairs?	^	4.22	0.84

A2*. Does your health now limit you in these activities? Walking 100 yards	**SF-36**	**4.09**	**0.85**

**A3. What degree of difficulty do you have walking long distances on the flat (greater than 1/2 mile)?**	**SF-36**	**4.06**	**0.89**

A4. What degree of difficulty do you have bending to floor?	WOMAC	3.63	1.02

A5. What degree of difficulty do you have climbing up and down one flight of stairs?	^	3.57	0.97

**A6. What degree of difficulty do you have putting on socks/stockings?**	**WOMAC**	**3.47**	**1.14**

**A7. What degree of difficulty do you have ascending stairs?**	**WOMAC**	**3.36**	**0.91**

A8. What degree of difficulty do you have rising from sitting?	WOMAC	3.32	0.84

**A9. What degree of difficulty do you have descending stairs?**	**WOMAC**	**3.31**	**0.95**

A10. What degree of difficulty do you have lifting?	AIMS	3.28	1.04

**A11. What degree of difficulty do you have standing?**	**WOMAC**	**3.27**	**0.93**

A12. What degree of difficulty do you have walking on the flat?	WOMAC	3.26	0.82

A13. What degree of difficulty do you have taking off socks/stockings?	WOMAC	3.24	1.13

**A14. Do you use a walking stick?**	**FLP**	**3.21**	**1.69**

A15. What degree of difficulty do you have rising from bed?	WOMAC	3.04	0.96

A16. What degree of difficulty do you have putting on/off shoes?	WOMAC	2.87	1.20

A17*. Does your health now limit you in these activities? Bending, kneeling or stooping	**SF-36**	**2.85**	**1.25**

A18. What degree of difficulty do you have getting on/off toilet?	WOMAC	2.72	0.99

A19. What degree of difficulty do you have lying in bed?	WOMAC	2.65	1.03

A20. What degree of difficulty do you have sitting?	WOMAC	2.56	0.93

A21. What degree of difficulty do you have dressing yourself (except shoes and socks)?	HAQ	2.15	0.98

A22. What degree of difficulty do you have washing and drying yourself?	SIP	2.13	1.01

A23. What degree of difficulty do you have washing your hair?	HAQ	1.91	1.06

A24. Do you need someone to help you go upstairs?	SIP	1.80	1.15

A25. Do you need someone to help you when you are walking?	SIP	1.78	1.01

**A26. Do you need someone to help you go downstairs?**	**SIP**	**1.78**	**1.17**

Items in bold removed by CTT/IRT item analysis

*These items had three categories and were rescaled to a five point scale.

^ Stair items: There was almost every combination of stair use represented in the original item pool. For parsimony not all combinations could be added at this stage, these two were added to complement and constrast with the stair items already in.

**Table 3:** P_ctt items ordered by difficulty

**Item**	**Origin**	**Mean**	**s.d.**
P1. How does your joint problem restrict your opportunities for leisure activities?	WHOQOL	3.82	0.94

**P2. How does your joint problem restrict you doing your hobbies?**	**FLP**	**3.41**	**1.19**

P3. How does your joint problem restrict you doing your usual social activities?	FLP	3.23	1.09

P4. How does your joint problem restrict you visiting friends or relatives?	AIMS	2.60	1.26

P5. How much of the time has your physical health or emotional problems interfered with your social activities (like visiting with friends)?	SF-36	2.54	1.30

**P6. How much do you enjoy life?**	**WHOQOL**	**2.36**	**0.76**

**P7. How healthy is your physical environment?**	**WHOQOL**	**2.28**	**0.86**

**P8. How available to you is the information that you need in your day-to-day life?**	**WHOQOL**	**2.06**	**0.85**

**P9. How satisfied are you with your personal relationship?**	**WHOQOL**	**2.06**	**0.99**

P10. How does your joint problem restrict you having friends or relatives over to your home?	AIMS	1.95	1.07

**P11. How satisfied are you with your transport?**	**WHOQOL**	**1.93**	**0.80**

P12. How does your joint problem restrict you getting on with people (friends and family)?	LHS	1.89	1.02

**P13. How satisfied are you with your access to health services?**	**WHOQOL**	**1.86**	**0.75**

**P14. How satisfied are you with the support you get from your friends?**	**WHOQOL**	**1.79**	**0.74**

**P15. How does your joint problem restrict how much money you have?**	**DRP**	**1.72**	**1.22**

P16. How does your joint problem restrict you affording things you need?	LHS	1.66	1.09

P17. How does your joint problem restrict you showing affection?	FLP	1.58	0.96

**P18. How satisfied are you with the conditions of your living place?**	**WHOQOL**	**1.58**	**0.72**

P19. How does your joint problem restrict you telephoning friends or relatives?	AIMS	1.26	0.62

*How does your joint problem restrict your capacity for work?'	**WHOQOL**	**n/a**	**n/a**

Items in bold removed by item analysis

*Item removed as greater than 10% missing data (no further analysis carried out)

#### Criterion measure for validation of new measures

The SF-36 subscales of pain (SF_pain), physical function (SF_phys) and social participation (SF_soc) were used as criterion variables for I, A & P respectively [1]. For all items a high score implies low limitation.

### Analysis

Initially, for both CTT and IRT, the frequency distribution of each I, A & P item was explored. Items with > = 10% missing data were excluded [35]. As the results from the CTT and IRT were to be compared, it was necessary to ensure that such analyses were based on the same data so subjects with missing data on either analysis were excluded.

#### CTT approach

The following six aspects of CTT were explored: a) Item difficulty was reported from the mean and standard deviation. An item with a large mean would indicate the sample is more limited on that item than on an item with a lower mean; b) An assumption for correlational methods is that the items have local independence i.e. there is no relationship between items controlling for the respondents position on the underlying construct. However, when the item pool was developed some items with overlapping content were retained in the initial item pool as there was no criteria on which to judge which items to retain or delete. These items would violate the assumption of local independence and so were grouped into independent sets (e.g. the four stair items were grouped into two independent sets of two items). The analyses were run separately using one of the sets and then repeated with the other set so as not to violate the assumptions. The results for each item set were compared to decide which items to retain; c) Pairs of redundant items were identified if they had very high correlations >0.87 (i.e.75% shared variance). The item, from the pair, that caused the greatest reduction in alpha if the item was deleted was retained; d) Internal reliability was examined using Cronbach's alpha. Items were deleted that would cause an increase in alpha if they were removed. The analysis was repeatedly rerun until no items were deleted; e) Item to Total Correlations (ITC) were calculated by removing the item from the hypothesised construct total and then correlating the item with that total (without the item). Items that had a low item to total correlation of <0.4 were deleted [34,36]; f) Multi-trait analysis (MAP) [37] was carried out to identify items that correlated higher with other I, A, P total(s) than with the total of the hypothesised construct minus the item with such items being deleted. The totals for each construct were based on the items that resulted from the earlier analysis. These totals were referred to as I_map, A_map and P_map.

Once all these steps had been completed for each construct, internal reliability, ITC and MAP analyses were rerun on the resultant sets of items

#### Item Response Theory approach

##### IRT model

For each construct Samejima's graded response model (GRM) [38] was fitted using MULTILOG [39]. The GRM is suitable for ordered polytomous responses and can deal with items that have a different number of response categories. The probability of a response to an item for a subject that has a trait level theta (θ) is both a function of the slope i.e. the discrimination (a) and the location parameters (b) that indicate the items difficulty. In a polytomous model there is more than one location parameter. The number of location parameters is the number of response categories minus one. These location parameters are thresholds that reflect the location where a participant is 50% likely to respond above the category threshold. Information functions were calculated for the total test (measure) and for each item at various levels of the underlying construct as suggested by Cooke et al. (1999) [40]. The item characteristic curves (ICC's) and information curves for each item were also explored (but are not reported).

##### Model fit

Model and item fit was evaluated by comparing the observed proportion of responses for each category, with the model predicted values obtained from the item parameters and the estimated latent trait distributions. The difference between these observed and expected values indicate how well the model predicts the actual item responses. It has been suggested that a difference between these values of less than 0.01 indicates very good fit [17].

##### Model assumptions

An assumption of IRT is that the items are measuring a unidimensional underlying construct. The factor structure for each construct was explored using exploratory factor analysis. Common criteria for acceptable unidimensionality are if > = 20% variance is explained in the first factor [41] or if the ratio of the first to second eigenvalue is 3:1 or 4:1(e.g. [40,42]). Both of these criteria were used and varimax rotation and principal axis factoring were carried out.

IRT models assume that there is local independence. It was known that some items in the item pool were not locally independent. So as not to violate the assumption, two models were fitted for each set of dependent items. The total information function, item information function and model parameters were compared to inform choice of which of the dependent items (or sets of items) to retain.

##### Item information and discrimination

Items were removed with low discrimination and low item information as they are probably not well related to the underlying construct [43]. There does not appear to be an agreed value for an acceptable discrimination. However, values have been suggested greater than one [14] to two [44]. Here, items were removed if they had a discrimination parameter of less than 1.25. This value was chosen so that items were not removed too early in the development process.

#### Combine CTT and IRT item information

The items that were removed as the result of CTT and IRT methods were compared and contrasted. Where both methods agreed the item was removed. If only one method suggested item removal then each item was reviewed individually. An initial exploration of properties of the resultant measures was carried out.

To examine the validity of the new measures, the correlation with subscales of the criterion variable (SF-36) should be as hypothesised i.e. SF-36 subscales pain, physical function and social participation should correlate more strongly with I, A & P respectively, than with the other SF-36 subscale totals. Cronbach's alpha should be at an acceptable level (i.e. >0.8) and IRT should indicate that the measure is reliable across the underlying construct. Reliability across the construct can be expressed in terms of the information function such that: Reliability = (1-[1/information]) with the standard error of measurement (SEM) = 1/[sqrt (information)]. Therefore, acceptable reliability (>0.8) is where the information is >5. The distribution of each measure should be approximately normal, to enable standard parametric statistical testing where the distribution is assumed to be normal. Skewness and kurtosis were examined using a conservative alpha level of 0.001 (z = +/- 3.29) as with large samples it is easy to achieve a significant skewness and kurtosis even with only small deviations from normality [35]. However, the main method of examining the distributions of the measures was through graphical examination as this is the most appropriate method for large samples [35].

## Results

For I and A there were no items with greater than 10% missing data. However, one P item *'How does your joint problem restrict your capacity for work?*', had 10% missing data and was dropped from the item pool.

Exploratory factor analyses were run for each set of items (I, A and P) to explore unidimensionality. Separate analyses were run with each dependent variable set, so as not to violate the assumption of local independence. All three sets of items had the ratio of their first to second eigenvalue >3. The ratio was highest for Impairment (6.7), then Activity Limitation (5.46 to 5.99) and then Participation Restriction (3.63 to 3.69). All three pools of items also had the first factor explaining >20% variance with Activity Limitation having the largest variance explained by the 1^st ^factor (>43%). There appeared to be acceptable evidence of a dominant first factor and, therefore, sufficient evidence of unidimensionality.

For ease of reading, the set of items entered into the first CTT analyses are referred to as I_ctt, A_ctt and P_ctt. The set of items entered into the first IRT analysis are referred to as I_irt, A_irt, P_irt. The resultant sets of uncontaminated items from the combination of both analyses are referred to as the Aberdeen

IAP measures (Ab-IAP) comprising Ab-I, Ab-A and Ab-P. The results for the CTT and IRT analysis are initially reported by construct and then the reliability and validity of final measures are explored together.

### A) IMPAIRMENT

#### Classical test theory approach

The mean item difficulties ranged from 2.90 to 4.21 [possible range 1–5] (see Table 1).

Two items were not locally independent, Item I6 '*Have you been troubled by pain from your joint in bed at night?*' and Item I10 '*Has pain from your joint kept you awake during your night-time sleep?*' as a positive answer to item I10 would imply a positive answer to item I6. Therefore, two separate analyses were run. Cronbach's alpha and ITC were higher with I6 (alpha = 0.867, ITC = 0.57) compared to item I10 '*Has pain from your joint kept you awake during your night-time sleep?*' (alpha = 0.865, ITC = 0.54) and so this latter item was removed.

The MAP analysis indicated that the Impairment item I2 '*What degree of difficulty do you have bending and rotating your affected joint?'*was more highly correlated with the A_map total (r = 0.65 p < 0.005) than with the I_map total without I2 (r = 0.53 p < 0.0005). The Impairment item I8 *'How severe is your stiffness after sitting, lying or resting later in the day' *was also more highly correlated with the A_map total = 0.55 p < 0.005) than with the I_map total without I8 (r = 0.54 p < 0.0005). Therefore items I2 and I8 were removed.

There were no redundant items, no items that increased Cronbach's alpha if the item was deleted and no ITC's < 0.4. There were no additional changes when all analyses were rerun with the resultant set of 10 Impairment items (Cronbach's alpha = 0.848).

#### Item response theory approach

Due to possible violations of the assumption of local independence, the items I6 '*Have you been troubled by pain from your joint in bed at night?*' and I10 '*Has pain from your joint kept you awake during your night-time sleep?' *were explored in separate analyses. The model with item I6, resulted in higher discriminating parameter, information and overall total information than the model with item I10. Therefore, the model with item I6 was retained and is now explored.

The I_irt items showed generally good discrimination (a > 1.25) except for one item I12 *'How often have you had pain in two or more joints at the same time?' *(a = 1.09). This item also had low information across the construct and was removed from the item pool. The information functions across the construct showed that the items were informative across the construct except at the highest end of the construct i.e. those with very high impairment. The item with the highest information and discrimination was I5 '*How active has your arthritis been?' *(see Table 4).

**Table 4:** I_irt item parameters

	**IRT item parameters**				
	**Discrim**	**Difficulty: location parameters**			

**I_irt item**	**a**	**b1** **(se)**	**b2** **(se)**	**b3** **(se)**	**b4** **(se)**

I1. Does remaining standing for 30 minutes increase your pain?	1.38	-4.25 (0.73)	-2.39 (0.29)	-1.22 (0.16)	-0.07 (0.11)

I2. What degree of difficulty do you have bending and rotating your affected joint?	1.46	-3.55 (0.47)	-2.31 (0.25)	-0.68 (0.12)	1.08 (0.14)

I3. How would you describe the pain you usually have from your joint?	2.33	-5.34 (-)	-2.47 (0.35)	-0.81 (0.09)	1.56 (0.13)

I4. How often have you had severe pain from your arthritis?	2.15	-2.82 (0.30)	-1.67 (0.15)	-0.56 (0.09)	1.21 (0.11)

I5. How active has your arthritis been?	2.50	-2.81 (0.31)	-1.94 (0.17)	-0.50 (0.08)	1.25 (0.11)

I6. Have you been troubled by pain from your joint in bed at night?	1.52	-2.65 (0.30)	-1.22 (0.15)	-0.45 (0.11)	0.75 (0.12)

I7. How severe is your stiffness after first wakening in the morning?	1.81	-2.88 (0.31)	-1.54 (0.15)	0.11 (0.09)	2.02 (0.19)

I8. How severe is your stiffness after sitting, lying or resting later in the day?	1.51	-3.62 (0.52)	-1.64 (0.19)	0.54 (0.11)	2.54 (0.27)

I9. How long has your morning stiffness usually lasted from the time you wake up?	1.34	-3.38 (0.43)	-1.05 (0.16)	0.65 (0.12)	1.57 (0.19)

I11. Have you felt that your knee or hip might suddenly 'give way' or let you down?	1.32	-2.62 (0.32)	-0.79 (0.14)	0.97 (0.14)	2.24 (0.25)

**I12. How often have you had pain in two or more joints at the same time?**	**1.09**	**-2.43** **(0.32)**	**-0.63** **(0.15)**	**0.76** **(0.15)**	**2.52** **(0.31)**

I13. Have you had any sudden, severe pain – 'shooting', 'stabbing' or 'spasms' – from the affected joint?	1.33	-2.98 (0.38)	-0.83 (0.14)	1.34 (0.17)	2.72 (0.31)

**TOTAL**					

Key: Items in bold = items with low discrimination parameter (< 1.25), (-) = not calculated

Thirteen items had all the differences between observed and expected response categories < 0.01, with only one item (I1) having one of the five response differences > 0.01 but less than 0.02. This analysis indicated very good fit.

#### Combining the IRT & CTT analyses

When the two dependent items were explored (I6, I10), both CTT and IRT suggested that the item I10 '*Has pain from your joint kept you awake during your night-time sleep?' *be removed from the item pool. Hence, this item was removed from the combined item pool.

Two items were removed by the CTT MAP analysis. One of the items, I2 '*What degree of difficulty do you have bending and rotating your affected joint?'*, was written as an attempt to convert a clinician measure of the degrees of of motion in the joint to a self-report item. The participants' responses indicate that it reflects Activity Limitation rather than Impairment.

The MAP analysis also suggested removal of item I8 '*How severe is your stiffness after sitting, lying or resting later in the day?' *This item was also be seen to be tapping Activity Limitation. Hence, it seemed appropriate to remove these two items from the combined item pool.

The final item identified for removal was I12 '*How often have you had pain in two or more joints at the same time?' *This was identified by IRT as having very low information and low discrimination. This item also had the lowest ITC from the CTT analysis and was removed from the combined item pool. Thus nine items were retained and four items removed (see Table 1 where items in bold were removed).

### B) ACTIVITY LIMITATION

#### Classical test theory approach

The mean item difficulties ranged from 1.78 to 4.22 (see Table 2).

There were two sets of items that may violate the assumption of local independence, 4 items concerning stairs and 3 items about walking. The four stair items were split into 2 independent sets: set (1) A7 *'What degree of difficulty do you have ascending stairs?' *and A9 *'What degree of difficulty do you have descending stairs?' *and set (2) A1 *'What degree of difficulty do you have climbing up and down **several **flights of stairs?' *and A5 *'What degree of difficulty do you have climbing up and down **one **flight of stairs?' *The three walking items were split into 2 independent groups set (3) A12 *'What degree of difficulty do you have walking on the flat?' *and set (4) A2 *'Does your health now limit you in these activities? Walking 100 yards?' *and A3 *'What degree of difficulty do you have walking long distances on the flat (greater than 1/2 mile)?' *Sets (2) and (3) led to higher Cronbach's alphas and ITC's and hence these sets were retained (see Additional file 2 for details).

The correlations between all the remaining items were examined for redundant items. Items with very high correlations (r = 0.881) were A6 *'What degree of difficulty do you have putting on socks/stockings?' *(Cronbach's alpha if item deleted = 0.937, ITC = 0.699) and A13 *'What degree of difficulty do you have taking off socks/stockings?' *(Cronbach's alpha if item deleted = 0.937, ITC = 0.704). The reliability statistics were very similar but A13 *'What degree of difficulty do you have taking off socks/stockings?' *performed slightly better so this was retained and item A6 was removed. Another high correlation (r = 0.995) was found between A24 '*Do you need someone to help you go upstairs?' *(Cronbach's alpha if item deleted = 0.939, ITC = 0.606) and A26 *'Do you need someone to help you go downstairs?' *(Cronbach's alpha if item deleted = 0.939, ITC = 0.591). Hence, item A26 was deleted.

There was an increase in Cronbach's alpha if two items were deleted and, hence, they were removed. These items were A14 '*Do you use a walking stick?*' and A17 '*Does your health now limit you in these activities? Bending, kneeling or stooping'*.

The MAP analysis indicated that one item, A11 *'What degree of difficulty do you have standing?'*, was more correlated with the I_map total (r = 0.598) than with the A_map total without A11 (r = 0.586) and was removed.

No remaining items had ITC < 0.4. There were no additional changes when all analyses were rerun with the resultant set of 17 Activity Limitation items (Cronbach's alpha = 0.939).

#### Item response theory approach

As in the CTT analysis, due to the assumption of local independence the sets of stair and walking items were analysed separately. Models with stair set (2) and walking set (3) resulted in higher discriminating parameter, information and overall total information compared to the models with the other sets of items (see Additional file 2 for details). Hence the model with A1, A5 and A12 and the 19 other items is now reported.

Twenty of the items had good discrimination (a > 1.25). However, 2 items (A14, A17) had low discrimination (a < 1.25) and low information across the construct. These items concerned using a walking stick and an item about bending, kneeling and stooping. These items were removed from the item pool.

The total and individual item information functions showed good information across the construct except at the lowest end of the construct i.e. those with very low activity limitation. The most discriminating and informative item was A15 '*What degree of difficulty do you have rising from bed?' *(see Table 5).

**Table 5:** A_irt item parameters

	**Item parameters**				
**A_irt item**	**Discrim**	**Difficulty: location parameters**			

	**a**	**b1** **(se)**	**b2** **(se)**	**b3** **(se)**	**b4** **(se)**

A1. What degree of difficulty do you have climbing up and down several flights of stairs?	1.72	-3.73 (0.59)	-2.62 (0.29)	-1.37 (0.14)	0.21 (0.10)

A4. What degree of difficulty do you have bending to floor?	1.91	-2.54 (0.25)	-1.58 (0.16)	-0.32 (0.09)	1.10 (0.12)

A5. What degree of difficulty do you have climbing up and down one flight of stairs?(*)	1.91	-2.76 (0.29)	-1.64 (0.15)	-0.07 (0.09)	1.13 (0.13)

A6. What degree of difficulty do you have putting on socks/stockings?	2.27	-1.87 (0.17)	-1.12 (0.11)	-0.03 (0.07)	0.96 (0.10)

A8. What degree of difficulty do you have rising from sitting?	2.07	-2.82 (0.34)	-1.41 (0.13)	0.34 (0.08)	1.82 (0.17)

A10. What degree of difficulty do you have lifting?	1.79	-2.17 (0.21)	-1.15 (0.12)	0.24 (0.09)	1.70 (0.17)

A11. What degree of difficulty do you have standing?	1.41	-2.90 (0.35)	-1.41 (0.17)	0.41 (0.12)	2.20 (0.26)

A12. What degree of difficulty do you have walking on the flat ?	1.47	-3.27 (0.41)	-1.57 (0.17)	0.52 (0.12)	2.42 (0.29)

A13. What degree of difficulty do you have taking off socks/stockings?	2.34	-1.79 (0.15)	-0.89 (0.10)	0.35 (0.07)	1.14 (0.11)

**A14. Do you use a walking stick?**	**0.95**	**-1.21** **(0.22)**	**-0.43** **(0.17)**	**-0.20** **(0.16)**	**0.63** **(0.18)**

A15. What degree of difficulty do you have rising from bed?	3.12	-1.68 (0.13)	-0.80 (0.08)	0.66 (0.07)	1.60 (0.11)

A16. What degree of difficulty do you have putting on/off shoes?	2.29	-1.29 (0.11)	-0.37 (0.09)	0.62 (0.08)	1.51 (0.12)

**A17. Does your health now limit you in these activities? Bending, kneeling or stooping**	**1.02**	**-4.52** **(1.24)**	**-1.76** **(0.34)**		

A18. What degree of difficulty do you have getting on/off toilet?	2.80	-1.36 (0.11)	-0.35 (0.07)	0.95 (0.08)	1.97 (0.16)

A19. What degree of difficulty do you have lying in bed?	2.21	-1.24 (0.11)	-0.30 (0.08)	1.17 (0.11)	2.23 (0.21)

A20. What degree of difficulty do you have sitting?	2.76	-1.19 (0.10)	-0.26 (0.07)	1.29 (0.10)	2.63 (0.27)

A21. What degree of difficulty do you have dressing yourself (except shoes and socks)?	2.71	-0.51 (0.08)	0.27 (0.07)	1.78 (0.13)	2.38 (0.23)

A22. What degree of difficulty do you have washing and drying yourself?	2.53	-0.43 (0.08)	0.24 (0.07)	1.70 (0.14)	2.83 (0.35)

A23. What degree of difficulty do you have washing your hair?	2.05	0.01 (0.08)	0.60 (0.09)	1.86 (0.17)	2.78 (0.32)

A24. Do you need someone to help you go upstairs?	1.63	0.21 (0.10)	1.18 (0.14)	1.64 (0.17)	2.23 (0.24)

A25. Do you need someone to help you when you are walking?	1.33	0.01 (0.11)	1.56 (0.20)	2.20 (0.27)	3.27 (0.44)

A26. Do you need someone to help you go downstairs?	1.59	0.25 (0.10)	1.24 (0.14)	1.66 (0.18)	2.12 (0.23)

**TOTAL**					

Key: Items in bold = items with low discrimination parameter (< 1.25).

Seventeen of the items had all differences between observed and expected response categories < .01 with only five items (A6, A15, A13, A18, A23) having one of the five responses > 0.01 but less than 0.02. This indicated overall good fit for the 22 retained items

#### Combining the IRT & CTT analysis

There were two sets of dependent items involving walking and stair use. Both methods suggested the removal of the same item set and so they were removed from the combined item pool.

Two items, A14 '*Do you use a walking stick?' *and A17 *'Does your health now limit you in these activities? Bending, kneeling or stooping'*, were removed from the combined item pool as they were identified by both methods. From CTT, this was indicated by alpha increasing when the item was deleted and the IRT indicated that both these items had low discrimination and low information across the construct (see Table 5). The latter of these items was asking about more than one activity limitation i.e. bending, kneeling and stooping and items that ask more than one question at the same time should be avoided as each limitation may be answered differently.

One item was identified by CTT MAP for removal A11 *'What degree of difficulty do you have standing?' *While this was not identified from the IRT, this item did have relatively low discrimination (a = 1.41) and information. This item was also different from almost all the other items as the other items involved body movement whereas this item did not. Considering all these findings, this item was removed from the combined item pool.

Two pairs of items were identified as having very high correlations (A6, A13 and A24, A26). The CTT indicated that A6 and A26 should be removed. The item parameters of the pairs of items were explored in the IRT analysis. This analysis identified the same item from each pair as the most appropriate for removal (see Table 5). The shape of Item Characteristic Curve (ICC) for each pair was almost identical with the item identified for removal having slighly lower information across the construct. Therefore, the identified items were removed from the combined item pool. This resulted in 17 items being retained and 5 items being removed (see Table 2 where items in bold were removed).

### C) PARTICIPATION RESTRICTION

#### Classical test theory approach

The mean item difficulties ranged from 1.26 to 3.82 (see Table 3).

There were two items with similar content and so may violate the assumption of local independence (and very high correlations (r = 0.885)). Item P15 *'How does your joint problem restrict how much money you have?' *(Cronbach's alpha = 0.874, ITC = 0.407) and P16 *'How does your joint problem restrict you affording things you need?' *(Cronbach's alpha = 0.877, ITC = 0.464). Hence, P15 was removed from the item pool.

Three items were removed as they had ITC < 0.4. These were P11 *'How satisfied are you with your transport?*' with ITC = 0.39; P13 *'How satisfied are you with your access to health services?' *with ITC = 0.30; P14 *'How satisfied are you with the support you get from your friends?' *with ITC = 0.33.

No redundant items were identified and no items were identified by the MAP analysis or from Cronbach's alpha. There were also no additional changes when all analyses were rerun with the resultant set of 15 Participation Restriction items (Cronbach's alpha = 0.875).

#### Item Response Theory Approach

Due to the assumption of local independence separate models were explored with Item P15 *'How does your joint problem restrict how much money you have?' *and P16 *'How does your joint problem restrict you affording things you need?' *Item P16 had better discrimination and total information than P15 and so the model with P16 is now reported.

Nine items (P2, P6, P7, P8, P9, P11, P13, P14, P18) had low discrimination and information and were removed from the item, pool. Six of these items originated from the WHOQOL (WHOQOL group, 1998). The item with the highest information and discrimination was P4 *'How does your joint problem restrict you visiting friends or relatives?' *(see Table 6).

**Table 6:** P_irt item parameters

	**Item parameters**				
**P_irt item**	**Discrim**	**Difficulty: location parameters**			

	**a**	**b1** **(se)**	**b2** **(se)**	**b3** **(se)**	**b4** **(se)**

P1. How does your joint problem restrict your opportunities for leisure activities?	1.39	-3.40 (0.41)	-2.19 (0.24)	-0.90 (0.13)	1.05 (0.16)

**P2. How does your joint problem restrict you doing your hobbies?**	**1.09**	**-2.54** **(0.32)**	**-1.54** **(0.21)**	**-0.30** **(0.13)**	**1.58** **(0.24)**

P3. How does your joint problem restrict you doing your usual social activities?	1.93	-2.16 (0.18)	-0.89 (0.10)	0.13 (0.09)	1.57 (0.16)

P4. How does your joint problem restrict you visiting friends or relatives?	2.84	-0.90 (0.08)	-0.03 (0.07)	0.67 (0.08)	1.80 (0.13)

P5. How much of the time has your physical health or emotional problems interfered with your social activities (like visiting with friends, relatives, etc.)?	2.16	-0.76 (0.09)	-0.11 (0.08)	0.79 (0.09)	1.72 (0.15)

**P6. How much do you enjoy life?**	**1.16**	**-2.69** **(0.36)**	**0.53** **(0.14)**	**2.62** **(0.35)**	**4.44** **(0.76)**

**P7. How healthy is your physical environment?**	**0.87**	**-2.17** **(0.35)**	**0.49** **(0.18)**	**3.31** **(0.56)**	**5.62** **(1.14)**

**P8. How available to you is the information that you need in your day-to-day life?**	**1.11**	**-1.25** **(0.19)**	**1.06** **(0.19)**	**2.81** **(0.39)**	**4.86** **(0.91)**

**P9. How satisfied are you with your personal relationship?**	**0.97**	**-1.10** **(0.20)**	**1.12** **(0.21)**	**2.45** **(0.37)**	**4.27** **(0.74)**

P10. How does your joint problem restrict you having friends or relatives over to your home?	1.94	-0.22 (0.08)	0.63 (0.10)	1.67 (0.16)	2.56 (0.28)

**P11. How satisfied are you with your transport?**	**0.91**	**-1.19** **(0.23)**	**1.87** **(0.33)**	**3.75** **(0.65)**	**5.40** **(1.12)**

P12. How does your joint problem restrict you getting on with people (friends and family)?	1.78	-0.19 (0.09)	0.66 (0.11)	1.95 (0.21)	3.05 (0.39)

**P13. How satisfied are you with your access to health **services?	**0.68**	**-1.35** **(0.32)**	**3.02** **(0.67)**	**5.14** **(1.16)**	**9.08** **(2.74)**

**P14. How satisfied are you with the support you get from your friends?**	**0.69**	**-1.08** **(0.28)**	**2.98** **(0.62)**	**6.35** **(1.46)**	**7.36** **(1.91)**

P16. How does your joint problem restrict you affording things you need?	1.26	0.61 (0.14)	1.35 (0.20)	2.11 (0.30)	2.99 (0.45)

P17. How does your joint problem restrict you showing affection?	1.42	0.50 (0.12)	1.34 (0.18)	2.38 (0.31)	3.49 (0.54)

**P18. How satisfied are you with the conditions of your living place?**	**0.97**	**-0.01** **(0.15)**	**2.91** **(0.49)**	**4.52** **(0.87)**	**6.55** **(1.68)**

P19. How does your joint problem restrict you telephoning friends or relatives?	2.27	1.08 (0.12)	1.80 (0.21)	2.97 (1.12)	4.77 (-)

**TOTAL**					

Key: Items in bold = items with low discrimination parameter (< 1.25), (-) = not calculated

Thirty two of the ninety (18 × 5) response categories had a difference between observed and expected response categories > 0.01 with 11 of these having a difference > 0.02. Therefore, the fit for Participation Restriction appears poorer than that of Impairment or Activity Limitation.

#### Combining IRT & CTT analysis

CTT identified three items with low ITC's (P11, P13, P14). These same three items were also identified as having low discrimination and information by the IRT analysis.

CTT also identified two items that were dependent and highly correlated (P15 and P16). The item P15 *'How does your joint problem restrict how much money you have?' *was identified for removal by CTT. IRT also identified this item as having low information and discriminatory ability compared to the other item in this pair. Hence, the item P15 was removed from the combined item pool.

IRT also identified six items with very low information and discriminating ability, that were not identified by the CTT. All of these items (except one) were derived from the WHOQOL [34]. These items may have had low information and discrimination with respect to measuring participation restriction as the WHOQOL was developed to explicitly measure quality of life, rather than particpation restriction (where quality of life was defined as '*'individuals' perception of their position in life in the context of the culture and value systems in which they live an in relation to their goals, expectations, standards and concerns' *[45]).

The other item with low information was concerned with hobbies (P2). This item may have been identified as a candidate for removal because the meaning of hobbies may not be clear or appropriate especially when other items include social and leisure activities i.e. what constitutes a hobby opposed to a leisure activity? Therefore, all 6 items identified from the IRT analysis were also removed from the item pool. Thus the CTT and IRT analysis resulted in 9 P items being retained and eleven items being removed including the one item already removed due to having greater than 10% missing data (see Table 3 where items in bold were removed).

### Resultant measures of I, A and P

The resultant measures of Impairment (9 items), Activity Limitation (17 items) and Participation Restriction (9 items) were explored. These uncontaminated measures are now referred to collectively as the Aberdeen Impairment, Activity Limitation and Participation Restriction measures (Ab-IAP) and individually as the Aberdeen Impairment measure (Ab-I), Aberdeen Activity Limitation measure (Ab-A) and the Aberdeen Participation Restriction measure (Ab-P).

Each of the uncontaminated measures correlated with the appropriate SF-36 subscale more than any other SF-36 subscale i.e. Ab-I with SF_pain; Ab-A with SF_phys and Ab-P with SF_soc (see Table 7).

**Table 7:** Pearson correlations of Ab-IAP with SF-36 subscales

	**SF_pain**	**SF_phys**	**SF_soc**
**Ab-I**	-.625(**)	-.515(**)	-.481(**)

**Ab-A**	-.604(**)	-.627(**)	-.596(**)

**Ab-P**	-.554(**)	-.541(**)	-.685^(**)/-.770(**)

** Correlation is significant at the 0.01 level (2-tailed).

^ As Ab-P contained an item based on an SF-36 item, this item was removed from the total of Ab-P.

All of the resultant measures had Cronbach's alpha > 0.8 (Cronbach's alpha Ab-I = 0.84 (n = 9), Ab-A = 0.94 (n = 17), Ab-P = 0.86 (n = 9).

**Figure 2:**
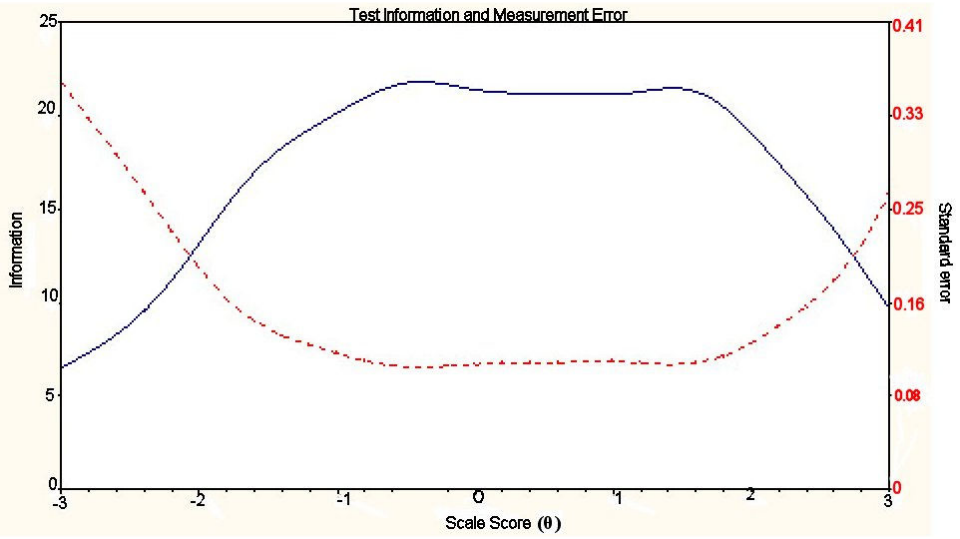
**Total information across the construct for Ab-A**. Test information curve - solid line; Standard error curve - dotted line.

**Figure 3:**
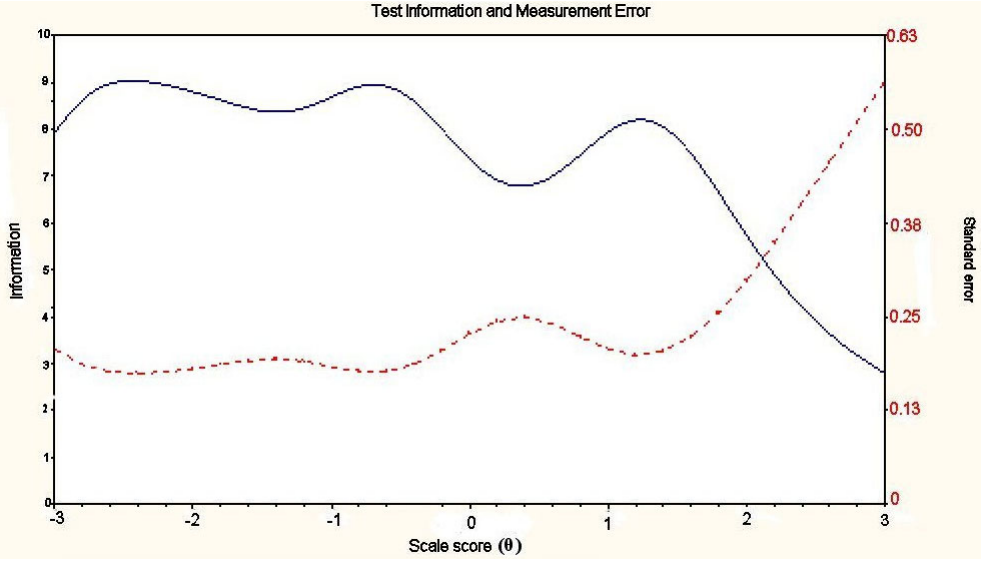
**Total information across the construct for Ab-I**. Test information curve - solid line; Standard error curve - dotted line.

**Figure 4:**
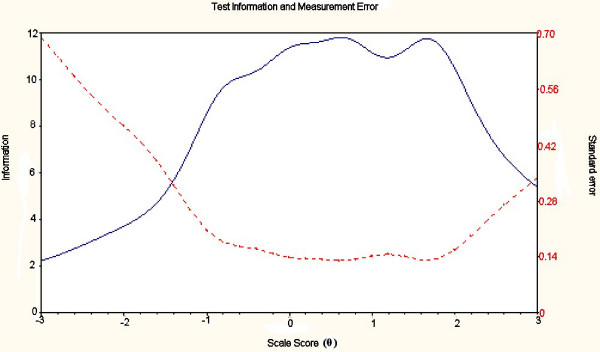
**Total information across the construct for Ab-P**. Test information curve - solid line; Standard error curve - dotted line.

The IRT analysis was rerun with the reduced items for each construct. The IRT indicated very good reliability across the whole construct for Ab-A (see Figure 2). All information was > 5 this equates to a reliability of > 0.80. There was good reliability across the central range of the construct for Ab-I and Ab-P (Figures 3 and 4). However, Ab-I was not adequately reliable at the very high levels of impairment (θ > 2) and the measure of Ab-P was not adequate at low levels of participation restriction (θ < 1.5). This suggests that new items should be added to address these areas.

There was very good fit for Ab-I with no differences between the observed and expected response categories > 0.01.

The fit for Ab-A indicated that 15 of the 85 response categories had differences between observed and expected response categories greater than 0.01, however, only one of these was greater than 0.02. This indicated reasonable fit but was worse than with all Activity Limitation items in the item pool.

The fit for Ab-P was improved over the fit with all the Participation Restriction items in the original item pool. Now, only 9 of the 45 differences were > 0.01. Seven of these were less than < 0.02 and the remaining two had a difference = 0.022. Six of these were from the first response category (i.e. the 'not at all' category). This was probably due to the positive skew on many of the Ab-P items.

**Figure 5:**
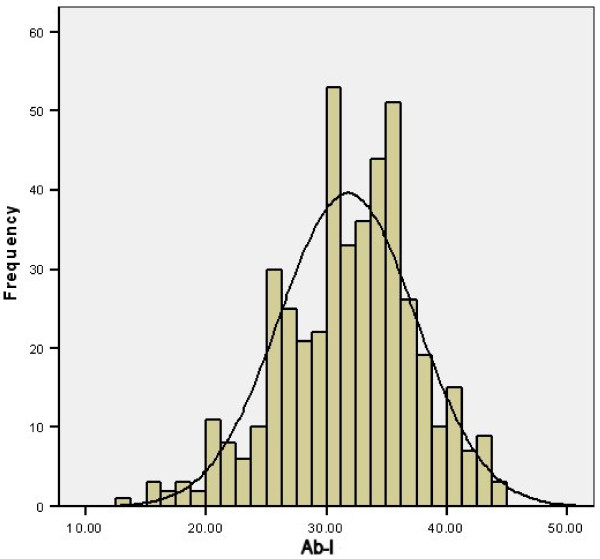
**Histogram of Ab-I**.

**Figure 6:**
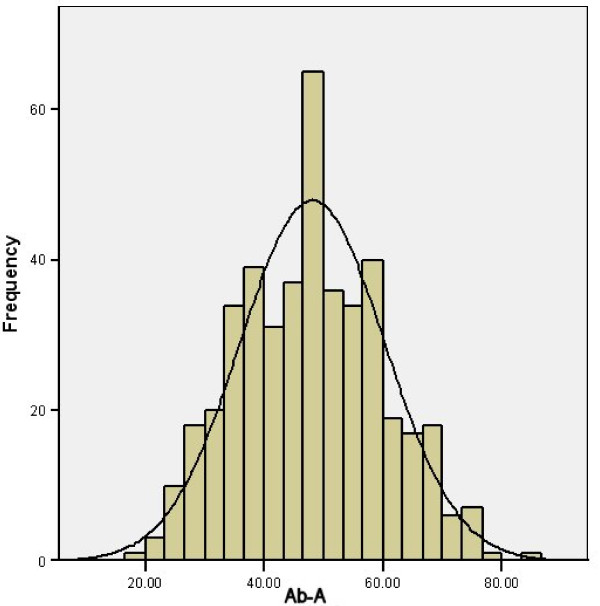
**Histogram of Ab-A**.

**Figure 7:**
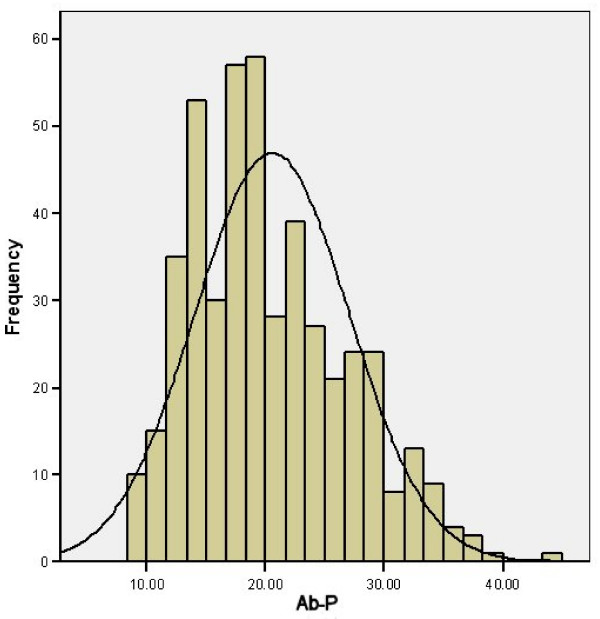
**Histogram of Ab-P**.

The distributions of Ab-I, Ab-A and Ab-P all appeared approximately normal when graphically examined (see Figures 5, 6 and 7). None of the other measures had significant skewness or kurtosis using an alpha level of 0.01.

## Discussion

In this paper, new measures of I, A and P have been developed that were specifically derived to measure each ICF component without contamination from other constructs in the model. These new measures can be used to improve assessment in both theory testing and the evaluation of interventions. For theory testing, the use of these uncontaminated measures should reduce over-inflation of observed relationships between constructs that may occur if measures are contaminated with other related constructs or the under-inflation that may occur if the measures are contaminated with constructs unrelated constructs. For example, the new measures should allow for more accurate evaluations of the relationships between the ICF components as these measures should not be contaminated with other constructs in the model.

For evaluating an intervention, the new measures allow for the assessment of the three distinct ICF components. Failure to adequately measure each distinguishable outcome might result in failure to detect benefit or harm due to an intervention or to a treatment. For example, in the treatment of patients with severe arthritis, an analgesic might predominantly affect impairment, an exercise programme might influence activity limitations and participation restrictions, but have little influence on impairment, whereas providing additional transport services might only alter participation restriction. If combined or contaminated measures are used then positive or negative effects may be masked.

While the previous work on the selection of items identified some items relevant for any population [1], this paper develops measures specifically in the context of joint replacement surgery, mainly for osteoarthritis. Thus the measures are particularly relevant for that population, even though some of the items originated from generic measures. Further work would be necessary to confirm the value of the measures for different populations.

Two methods of item analysis were explored, the traditional CTT approach and the more recent IRT method. These methods have their strengths and weaknesses. The use of both methods may yield more information than only using one of the methods. Each method was explored individually and then the results from each method compared and contrasted. CTT and IRT methods identified common items for removal from the item pool. Each method also suggested some items that could be removed that were not indicated by the other method using the criteria outlined. The CTT-MAP analysis indicated that three items were more highly correlated with a total other than the hypothesised construct total. There were feasible explanations for the removal of all three items. IRT additionally highlighted items that had low information and could possibly be removed. This was preferable to the CTT approach of item reduction where factor analysis is used and may result in small areas of a construct being covered. This problem is even more likely if some of the items have similar wordings as these would be the strongest indicator of the factor and be retained ahead of other items. Using IRT can also result in the items representing a small area of the construct. However, this is driven by a different theoretical approach to CTT, based upon items not discriminating well or not having much information.

The decision to use a discriminating parameter of < 1.25 as a criteria for item removal was somewhat arbitrary. As described earlier, the decision was based on published suggestions but as yet there is no consensus on what values for the discrimination parameter or information function are acceptable. Again, there were plausible reasons why items had been identified as having low information and so they were also removed from the item pool.

The IRT analysis indicated that the model fitted using the pool of candidate items for P_irt had poorer fit than the I_irt and A_irt models. However, as there is no consensus about how to assess model fit or how to deal with misfitting data [46], the effect of this is difficult to quantify and so this may have an effect on the results for Participation Restriction. The P_irt had fewer items than the I_irt or A_irt sets of items. This reflected the observation that commonly used measures in OA tended to focus on I and A. Our analysis of 342 items found only 44 pure P items[1]. Nevertheless, the resultant measure of Participation Restriction appeared to have acceptable properties.

The item analysis resulted in the removal of 4 Impairment items, 9 Activity Limitation items and 11 Participation Restriction items with 14 of these items being identified by both CTT and IRT. The resultant measures consisted of 9 Impairment items (Ab-I), 17 Activity Limitation items (Ab-A) and 9 Participation Restriction items (Ab-P). The correlations of the resultant measures with the criterion variable of the SF-36 appeared to follow the expected pattern. The measures had acceptable Cronbach's alpha (all > 0.8). However, when this was explored in more detail using IRT, Ab-I was not reliable at very high levels of impairment while Ab-P was not reliable at the low end of the construct. This suggests that new items should be written to cover these areas if it is to be used for all ability levels. So, for Ab-I, some 'easy' items should be written to discriminate the high end of the construct e.g. *'my joint is uncomfortable (never to always aches)'*. For Ab-P some new 'hard' items should be added to discriminate this area of the construct e.g. *'are you able to participate in sporting activities?' *This illustrates an advantage of using IRT, as the lack of reliability at the extremes of the construct was not identified by the CTT analysis. It is possible that the lack of reliable items at the ends of the I and P constructs may be due to the items having been selected from measures that were developed using CTT methods. For example, a high Cronbach's alpha can be achieved by selecting items that are all strongly related to each other but may cluster around a small area on the underlying construct. The total information was greatest for Ab-A with information > 10 across most of the construct.

The Graded Response Model fit was acceptable for the Ab-I, Ab-A and Ab-P models. The model fit was better than it had been for the candidate item models for Impairment (I_irt) and Participation Restriction (P_irt) but a little worse for Activity Limitation (A_irt). The distributions appeared approximately normal when graphically examined, although Ab-P had statistically a slight skew.

A two parameter IRT model was selected in order to be able to estimate both a difficulty and discrimination parameter. There is much debate between using the single parameter Rasch model (where item difficulty is estimated and equal item discrimination is assumed) or a more general 2 parameter IRT model. Some favour the single parameter Rasch model as they believe it adheres to the fundamental measurement principle that all items behave in the same way (i.e. the data must fit the model) [47]. Others favour using an IRT model that best fits the data and suggest the Rasch model may be too restrictive and can lead to discarding useful items (see [48,49]). In this study, we are interested in developing measures that are tailored to OA. We therefore chose to use an approach that allows us to select items that convey the most information about our chosen population rather than force particular properties on each item in our measure. In addition, with a limited set of items it is unlikely that sufficient items would be found that cover the construct as well as all having the same discrimination. The formation of very large item banks for computer adaptive testing (CAT), may, in the future, allow the use of the Rasch model to develop tailored questionnaires. Until such time, we take the pragmatic approach and select the two parameter IRT model.

The selected items could be explored further. If a shorter measure was required, stricter criteria could be used for selecting items with IRT. Alternatively, a decision could be made on how many items the resultant measure should have. Using IRT methods, items could be identified that have information (precision) across the construct domain [50].

The response rate of 43% was quite low but reasonable given the long length of the questionnaire (27 pages, 254 items). It appeared that the sample was representative as there were no differences between the responders and non-responders on gender, age and disability. The question remains to whether the 60% who did not participate were significantly different from the sample on other unmeasured variables.

This study was based on a population with severe hip or knee problems as they were assessed prior to surgery. If a measure is required to assess patients post-operatively, or patients in the earlier stages of osteoarthritis, then the same items should be useful as IRT is an invariant method (i.e. item parameters should be similar even with a sample that has different levels of 'ability'). However, the accuracy of the parameter estimates does depend on the limitation levels of the calibration sample. As the sample of patients about to undergo joint replacement has relatively low levels of 'ability' then the parameter estimates would be most accurate for the easier items. Hence, it would be useful to repeat the analysis on patients after surgery as these patients would have more 'ability' and thus should provide more accurate parameter estimates for the harder items. Additionally, this would also allow an empirically evaluation of the invariant property of IRT.

The resultant measures appeared to have acceptable properties to date. However, only a preliminary psychometric evaluation of reliability and validity was carried out. As reliability and validity can never be proved but is based on an accumulation of evidence, much further empirical testing needs to be carried out.

The resultant measures have been constructed to represent the theoretical constructs without contamination from other constructs in the ICF model to allow for the testing of the ICF model. However, this representation was based on the DCV judgements of expert judges and may not represent the discrimination made by respondents to the measures. It will be important to explore if the measures are statistically independent using patients responses to the items.

## Conclusion

These analyses have resulted in new measures that reflect the three ICF constructs (I, A and P) in people having joint surgery for severe arthritis. The new measures have good psychometric properties, discriminate well across the dimension and retain only informative, non-redundant items. While these measures can be improved further, they offer an advance on existing osteoarthritis measures in assessing ICF constructs.

The use of both CTT and IRT for item analysis appeared to provide more information than the use of only one of these methods. On preliminary exploration of the properties, the new measures appeared acceptable. However, additional items should be considered to cover the extreme ends of the construct for the impairment and participation restriction measures if a measure is required that covers the entire underlying construct.

## Competing interests

The authors declare that they have no competing interests.

## Authors' contributions

BP participated in the conception and design of the study, the analysis and the drafting and revision of the manuscript. MJ participated in the conception and design of the study and the drafting and revision of the manuscript. PD and DD contributed to the interpretation of the data and revision of the manuscript. All authors read and approved the final manuscript.

## Supplementary Material

Additional file 1 **Additional file 1: Initial item pool reduction**. Details of the initial item pool reduction. Click here for file

Additional file 2 **Additional file **1**: Local independence analysis for Activity Limitation**. Details of CTT and IRT local independence analysis for Activity Limitation. Click here for file
