# Supplementary material for: Measuring the ICF components of impairment, activity limitation and participation restriction: an item analysis using classical test theory and item response theory
Source: Health Qual Life Outcomes. 2009 May 7;7:41. doi: 10.1186/1477-7525-7-41 (PMC2696439; doi:10.1186/1477-7525-7-41)
Supplement: Additional file 1 — Initial item pool reduction. Details of the initial item pool reduction. [file 1477-7525-7-41-S1.doc]

**Additional file 1: Initial item pool reduction**

The pool of pure items comprised 74 I, 88 A and 44 P items. An initial procedure was necessary to eliminate items with overlapping content and reduce patient burden. This was carried out by ranking items in descending order by the t-values from the DCV for each construct [1] i.e. the highest ranked item for Impairment was rated as being the Impairment item that statistically, the judges were most confident in classifying to the I construct.

The items were reduced based on the following criteria for each construct: (a) the 10 items with the highest t-values were selected that had non-overlapping content, (b) other items that had a content area not covered by the top 10 items were selected (c) if two items represented the same content, the higher ranked item was selected. This procedure resulted in 13 I, 26 A and 20 P candidate items.

The A and P items were rewritten to have a consistent item format where feasible. The format for the activity limitation items was based on the WOMAC wording and response format i.e. ‘What degree of difficulty do you have ……’ and used a five point response format (none to extreme). The participation restriction items had the general format ‘How does your joint problem restrict you……’ with a five point response format (not at all to extremely). A consistent wording format was not feasible for the I items but they also had five point response format. Items that were from measures that were included in their entirety in the questionnaire (i.e. SF-36 and WHOQOL items were left in their original form to avoid asking the same item twice). The response formats were a five point scale, except for 2 items left in their original format of 3 response categories. Where necessary, items were recoded so that for all items a high score implied high limitation. Additionally, an Impairment item about pain keeping patient awake at night was added as this was not covered by the existing items although it had been identified by the research steering group as being a key question that determined recommendation for surgery (see Tables 1, 2 and 3 for each item and its origin).
