# Supplementary material for: Measuring the ICF components of impairment, activity limitation and participation restriction: an item analysis using classical test theory and item response theory
Source: Health Qual Life Outcomes. 2009 May 7;7:41. doi: 10.1186/1477-7525-7-41 (PMC2696439; doi:10.1186/1477-7525-7-41)
Supplement: Additional file 2 — Additional file 1: Local independence analysis for Activity Limitation. Details of CTT and IRT local independence analysis for Activity Limitation. [file 1477-7525-7-41-S2.doc]

**Additional file 2: Local independence: Activity Limitation items**

Four items concerning stairs and 3 items about walking were not independent. The four stair items were split into 2 independent sets: set (1) A7 *‘What degree of difficulty do you have ascending stairs?’* and A9*‘What degree of difficulty do you have descending stairs’* and set (2) A1*‘What degree of difficulty do you have climbing up and down* ***several*** *flights of stairs?’* and A5*‘What degree of difficulty do you have climbing up and down* ***one*** *flight of stairs?’* The three walking items were split into 2 independent groups set (3) A12 *‘What degree of difficulty do you have walking on the flat?’* and set (4) A2 *‘Does your health now limit you in these activities? Walking 100 yards?’* and A3 *‘What degree of difficulty do you have walking long distances on the flat (greater than ½ mile)?’*

CTT approach

Stair items: Reliability analyses comparing the two stair sets were carried out with each of the walking sets so as not to compromise the assumption of independence. The reliability with stair set (1) and walking set (4) (Cronbach’s alpha=0.940, ITC=A7-0.63, A9-0.59) indicated this set had slightly worse properties than an analysis with stair set (2) and walking set (4) (Cronbach’s alpha=0.941, ITC=A7-0.63, A9-0.69). The analysis was repeated with walking set (3) and produced similar results. Hence, stair set (1) (item A7 and A9) was removed from the item pool.

Walking items: Reliability analysis with walking set (3) (Cronbach’s alpha=0.941, ITC=0.57, no. of items=22) had very similar properties to walking set (4) (Cronbach’s alpha=0.941, ITC=A2-0.53, A3-0.58, no. of items=23). However, as walking set (3) only had one item, the reliability analysis was based on one less item than walking set (4) and as Cronbach’s alpha increases with item length it appeared appropriate to remove walking set (4) (items A2 and A3) from the item pool.

**IRT approach:**

As there were also dependent walking items, based on the CTT analyses the A12 ‘walking on the flat’ item was included at this stage. The dependent walking items were addressed after the stair items had been explored. The stair items set (2), A1 *‘What degree of difficulty do you have climbing up and down* ***several*** *flights of stairs?’* and A5 *‘What degree of difficulty do you have climbing up and down* ***one*** *flight of stairs?’* resulted in higher discriminating parameter, information and overall total information compared to the set (1) of the stair items A7 *‘What degree of difficulty do you have ascending stairs?’ and* A9 *‘What degree of difficulty do you have descending stairs?’* The analyses were repeated with the alternative set of walking items and suggested the removal of the same 2 stair items (A7 and A9), thus the model with these items was retained.

Dependent walking items: The dependent sets of walking items were compared. The analysis were first run with the stair set suggested from the previous analysis (i.e. using A1 *‘What degree of difficulty do you have climbing up and down* ***several*** *flights of stairs?’* and A5 *‘What degree of difficulty do you have climbing up and down* ***one*** *flight of stairs?’)* The analysis with the item A12*‘What degree of difficulty do you have walking on the flat?’* resulted in higher discriminating parameter, information and overall total information compared to the items A2 *‘Does your health now limit you in these activities? Walking 100 yards?’* and A3 ‘*What degree of difficulty do you have walking long distances on the flat (greater than ½ mile)?’* The analyses were also repeated with the other stair set. All analyses resulted in the same items sets being retained or removed.
